# A Flexible Optical pH Sensor Based on Polysulfone Membranes Coated with pH-Responsive Polyaniline Nanofibers

**DOI:** 10.3390/s16070986

**Published:** 2016-06-27

**Authors:** Nedal Abu-Thabit, Yunusa Umar, Elaref Ratemi, Ayman Ahmad, Faraj Ahmad Abuilaiwi

**Affiliations:** 1 Department of Chemical and Process Engineering Technology, Jubail Industrial College, Jubail Industrial City 31961, Saudi Arabia; umar_y@jic.edu.sa (Y.U.); Ratemi_e@jic.edu.sa (E.R.); Mohammad_aa@jic.edu.sa (A.A.); 2 Department of Chemistry, College of Science, University of Hafr Al Batin, Hafr Al Batin 31991, Saudi Arabia; faraj@uohb.edu.sa

**Keywords:** optical pH sensor membrane, flexible pH sensor, polysulfone, polyaniline, chemical oxidative polymerization, nanofibers

## Abstract

A new optical pH sensor based on polysulfone (PSU) and polyaniline (PANI) was developed. A transparent and flexible PSU membrane was employed as a support. The electrically conductive and pH-responsive PANI was deposited onto the membrane surface by in situ chemical oxidative polymerization (COP). The absorption spectra of the PANI-coated PSU membranes exhibited sensitivity to pH changes in the range of 4–12, which allowed for designing a dual wavelength pH optical sensor. The performance of the membranes was assessed by measuring their response starting from high pH and going down to low pH, and vice versa. It was found that it is necessary to precondition the sensor layers before each measurement due to the slight hysteresis observed during forward and backward pH titrations. PSU membranes with polyaniline coating thicknesses in the range of ≈100–200 nm exhibited fast response times of <4 s, which are attributed to the porous, rough and nanofibrillar morphology of the polyaniline coating. The fabricated pH sensor was characterized by a sigmoidal response (R^2^ = 0.997) which allows for pH determination over a wide dynamic range. All membranes were stable for a period of more than six months when stored in 1 M HCl solution. The reproducibility of the fabricated optical pH sensors was found to be <0.02 absorption units after one month storage in 1 M HCl solution. The performance of the optical pH sensor was tested and the obtained pH values were compared with the results obtained using a pH meter device.

## 1. Introduction

Polymeric membranes have been widely utilized as immobilization matrices for various types of sensors, including optical chemical sensors [1,2], biosensors [3,4] potentiometric sensors [5,6], and amperometric sensors [7,8]. Solid polymeric membrane supports offer advantages such as fast response time, minimal calibration requirement, flexibility, low cost and possibility for deposition into various types of substrates [9]. The performance of polymeric membranes can be enhanced by surface functionalization/grafting with hydrophilic/hydrophobic sites for obtaining maximum interaction with specific analyte [5,10]. Polysulfone (PSU) is an engineering thermoplastic polymer with high thermal and mechanical stability, transparency and resistance to many solvents and chemicals [11]. PSU has been employed as membrane matrix/support for fabrication of different types of sensors such as humidity [12,13], optical humidity [14], optical oxygen [15], enzyme biosensor [16] and strain sensors [17].

Optical sensors have been employed commercially for the determination of pH, heavy metal ions, CO_2_ and O_2_ gases and clinical parameters such as glucose and bacteria in blood [18]. Optical pH sensors are used for measurement and control of pH for applications in chemistry [10], biochemistry [19], clinical chemistry [20] and environmental sciences [21]. Planar and fiber-optic based sensing platforms have been used for fabrication of optical pH sensors with different geometries [22]. The change in the optical properties of the immobilized reagents/dyes is measured by recording the corresponding absorbance [23,24] or fluorescence [25,26,27] signal at certain pH conditions.

Optical pH sensors can be prepared by immobilization of pH-sensitive indicator dyes into a hydrophilic supporting polymer [23,25,28] or sol-gel matrix [22,29,30]. However, optical pH sensors prepared by the former method suffer from non-linear responses, narrow dynamic range for pH measurement and short lifetimes due to the leaching out of the immobilized indicator dyes. The shortcomings of the indicator-based optical pH sensors can be alleviated by using pH-responsive electrically conducting polymers (ECPs) such as polypyrrole and polyaniline [31,32]. Thin films coated with ECPs represent an interesting alternative to indicator-based pH sensor films due to their inherent optical response properties and wider dynamic range for pH measurement. Compared to leachable indicator dyes, water-insoluble ECPs are less susceptible to leaching which provides improved long-term stability and reproducibility for the fabricated optical pH sensor.

The pH-responsive nature of polyaniline has been utilized for fabrication of a layer-structured nanocomposite film for application as a smart pH self-adjusting switching system with high contrast ratio [33]. A novel optical pH sensor was fabricated using fluorescent beads coated with polyaniline [34]. The fluorescence emitted by the dye-doped polymer nanobeads is modulated by the thin conductive polyaniline coating, which responses to the variation in pH through visible-light absorption at pH values around 7.

Different forms of polyaniline have been used for fabrication of optical pH sensors such as dispersed polyaniline nanoparticles doped with poly(styrene sulfonic acid) [35], polyaniline-polyvinyl alcohol (PANI-PVA) composite membranes doped with formic acid [36], optically active polyaniline nanofibers doped with (*S*)-naproxen [37] and polyaniline nanofibers grafted onto the surface of polydimethylsiloxane microfluidic channels for dynamic pH imaging and mapping applications [38].

In this paper, we report on a novel, simple and cost effective method for the preparation of an optical pH sensor based on polyaniline-coated polysulfone (PANI-coated PSU) membranes with a well-defined pH response and reproducibility in the pH range of 4–12. Polyaniline functions as the pH-responsive conducting polymer which is coated on the PSU support to construct the flexible pH-responsive sensor membrane.

## 2. Experimental Section

### 2.1. Materials

PSU (35 kg/mol), aniline (ACS reagent, ≥99.5%), ammonium persulfate (APS) (ACS reagent, 98.0%), NaH_2_PO_4_·2H_2_O (ACS reagent, 98%–100.5%), Na_2_HPO_4_ (ACS reagent, 98%–102%), NaCl (>99.5%), Na_2_CO_3_ (ACS reagent ≥99.5%), and NaHCO_3_ (ACS reagent ≥99.7%) were received from Aldrich (Milwaukee, WI, USA). Aniline was distilled twice and stored in the dark at 5 °C before use.

The PBS buffer system was prepared as follows: 6.2400 g of NaH_2_PO_4_·2H_2_O (40 mmol) and 5.2596 g of NaCl (90 mmol) were dissolved in deionized water (1.0 L) with a total ionic strength of 130 mM. 5.6800 g of Na_2_HPO_4_ and 0.5844 g of NaCl were dissolved in deionized water (1.0 L) with a total ionic strength of 130 mM. Various buffers in the pH range 4–12 were prepared by the addition of equal amounts of the above two solutions, followed by adjusting the pH with a dropwise addition of 1 M HCl or 10% NaOH solutions. The pH of the prepared PBS buffers was measured using a pH meter device (Model 3510-JENWAY, Essex, UK) which was calibrated with three pH standard buffer solutions (4, 7 and 10).

### 2.2. Preparation of PANI-Coated PSU Sensor Membranes

Polysulfone membranes were casted from 10 wt % chloroform solutions using glass Petri dishes to yield membranes with thicknesses of ≈250 μm. The in situ chemical oxidative polymerization (COP) was carried out by placing PSU membranes into various concentrations of aniline/1 M HCl solutions, followed by addition of APS/1 M HCl solutions (Table 1). After a certain period of reaction time, the resulted PANI-coated PSU membranes were removed and washed repeatedly with methanol and 1 M HCl to ensure the removal of unreacted monomer, oligomers and excess initiator species. Precipitated polyaniline from the same reaction was collected, washed, dried at room temperature and used for FTIR and SEM analysis.

### 2.3. Sensor Characterization and pH Measurements

A double beam spectrophotometer Cintra 2020 (GBC Scientific Equipment, Melbourne, Australia) was used for recording the absorbance in the range of 400–1000 nm. The reference cell holder was filled with the desired pH buffer solution, and the sensor membrane (5 cm long × 0.8 cm wide) was placed and fixed in the sample cell holder filled with the same pH buffer solution and the measurements were performed in a batch mode. All the measurements were done as triplicates and the standard deviations were calculated accordingly. To calculate the pH values based on the detected absorbance, the optical pH sensor membrane was calibrated using a Four Parameter Logistic (4PL) nonlinear regression model, with sigmoidal curve fitting according to Equation (1):

(1) $$A\;=\;d\;+\;\frac{a\;-\;d}{1\;+\;{\left(\frac{pH}{c}\right)}^{b}}$$

where, ***A*** is the measured absorbance; a is minimum asymptote; *d* is the maximum asymptote; *c* is the inflection point; and *b* is the hill’s slope.

This enables the measurement of pH over a wide proton concentration range, as the pseudolinear part of the calibration curve is not the only part used. From the constructed calibration curve, the ***pH*** can be calculated as shown in Equation (2):

(2) $$pH=c\;x\;{\frac{\left(A\;-\;d\right)}{\left(a\;-\;A\right)}}^{\left(1/b\right)}$$

### 2.4. Instrumentation

Infrared spectra of pure polyaniline powder, PSU membranes and PANI-coated PSU membranes were recorded using IR Prestige-21 (Shimadzu, Kyoto, Japan) instrument; 32 scans were collected for each sample in the range of 400–4000 cm^−1^. The morphology of the PANI-coated PSU membranes was investigated by scanning electron microscopy (SEM), using a model MIRA 3 LMU instrument (TESCAN, Brno, Czech Republic). Individual membranes were mounted on aluminum specimen stubs with double-sided carbon adhesive tape and sputter-coated with a gold layer to reduce the buildup of charges on the surface of the sample. The obtained SEM images were analyzed by using the Tescan Lyra 3 software. Optical images were obtained by using a model BX51 optical microscope (Olympus, Sendai, Japan) equipped with a 5 Megapixel digital camera (NIKON, Sendai, Japan). The obtained optical images were analyzed by using Olympus Stream image analysis software.

## 3. Results and Discussion

### 3.1. Optimization of the Reaction Conditions

**Scheme 1:**
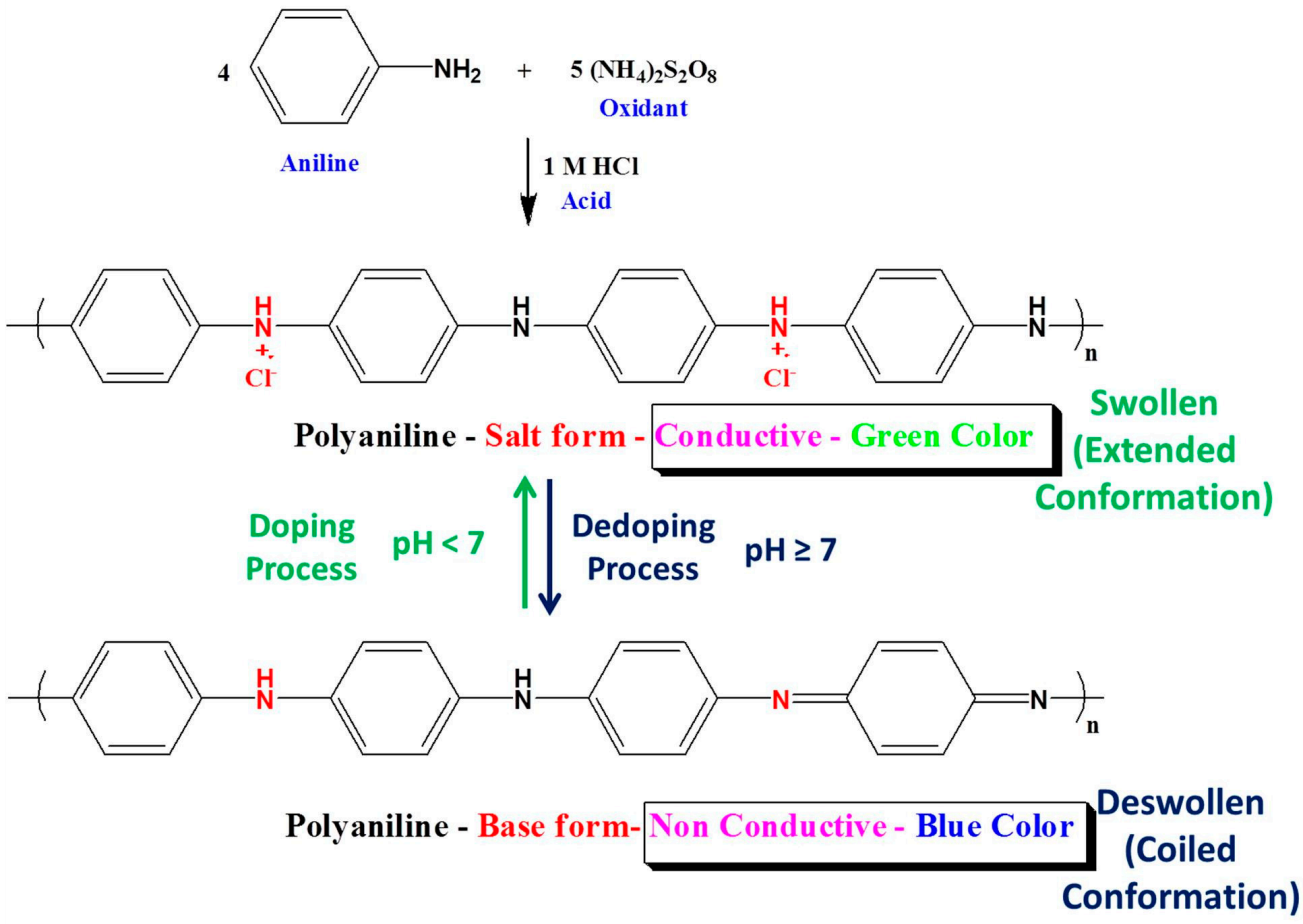
Chemical Oxidative Polymerization (COP) of aniline.

Chemical oxidative polymerization represents an effective, simple, cheap and quick method for coating polyaniline into hydrophilic, hydrophobic, conductive and non-conductive substrates with different geometries [39,40,41]. PSU membranes were selected as support due to: (1) their good mechanical properties; (2) transparency; and (3) negligible water absorption. The superior mechanical properties and transparency allow for fabrication of flexible optical sensor membranes with tunable thicknesses. The negligible water absorption helps to prolong the lifetime of the membrane during storage and use in acidic/basic aqueous solutions. In the current study, PSU membranes were coated with polyaniline by the in situ COP of aniline monomer in the presence of ammonium persulfate (APS) as redox initiator (Scheme 1). After the coating process, the transparent PSU membranes turned green due to the precipitation of conductive polyaniline onto the surface of PSU membranes. As shown in Table 1, the absorbance of the coated membranes increased with increasing the reaction time. A 10 min reaction time provided an absorbance of 0.63 absorption units, whereas the response obtained by doubling the reaction time was ≈1.1 absorption units. Hence, PSU membranes that reacted with aniline for 20 min were selected for further study as optical pH sensors.

### 3.2. Thickness of Polyaniline Coatings

One of the most interesting properties of polyaniline is its ability to form strongly adhered films into the surface of different substrates [42]. The in-situ polymerization is a suitable method for fabrication of well-defined thin films [43] The in-situ deposited polyaniline thin films obtained from COP exhibit thicknesses of <1 μm [44,45,46,47]. The thickness of the polyaniline coating can be controlled by variation of reaction time to produce films with thicknesses in the range of 180–250 nm [44]. Hence, in the current study the reaction time was varied at constant and dilute concentrations of both monomer and initiator. The low concentration of reactants promotes the formation of polyaniline nanofibers [48,49]. Although it was reported that the monomer: initiator ratio does not have a pronounced effect on the obtained film thickness [50], we used stoichiometric ratio, as shown in Scheme 1, to ensure the complete polymerization of monomer molecules during the course of polymerization.

The thickness (*d*) of polyaniline coating can be estimated by correlation with the absorbance for the green Emeraldine Salt at 400 nm (*A_400_*) using Equation (1) [45,51,52]:

*d* (*nm*) = 185 *xA_400_* (3)

As shown in Table 1, the estimated thicknesses of the formed films after 10 and 20 min were 101 ± 7 nm and 160 ± 9 nm, respectively. These results are very close to those thicknesses reported for PANI-coated glass films using similar reaction conditions [45,51].

### 3.3. FTIR Analysis

**Figure 1:**
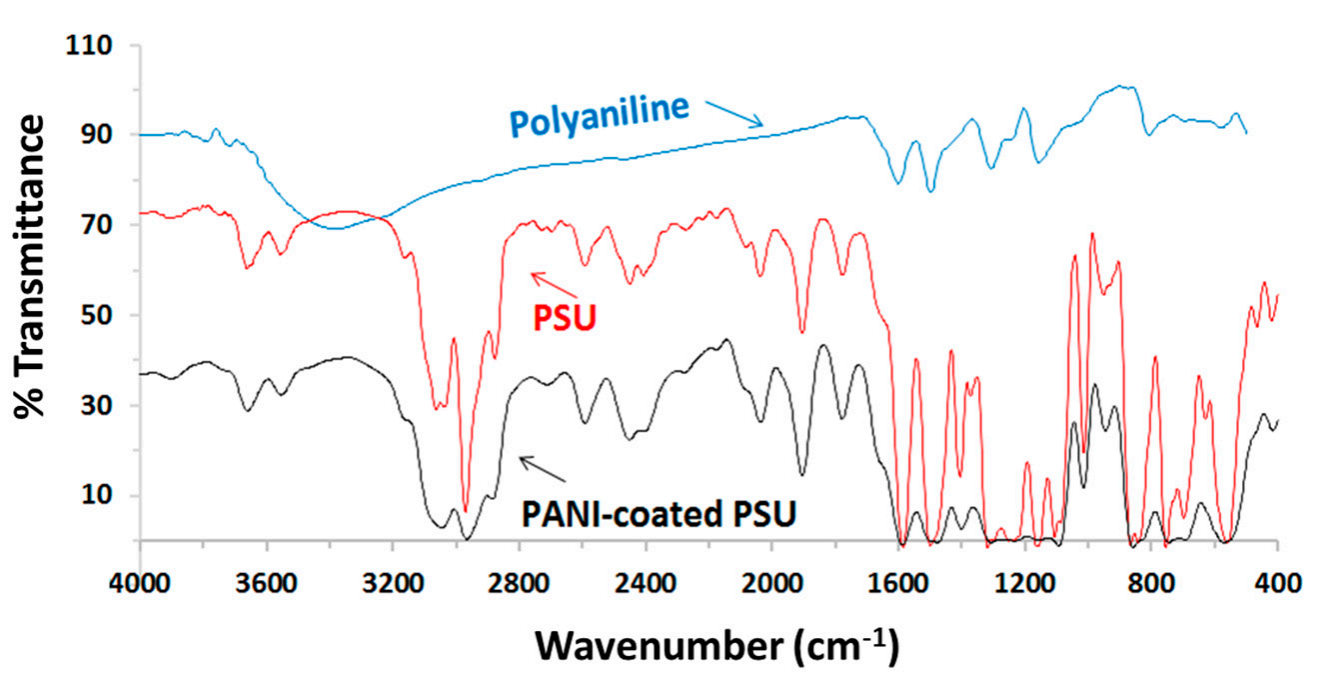
FTIR spectra for pure PANI in the acid form, pure PSU and PANI-coated PSU in the acid form.

Figure 1 shows the FTIR spectra for PANI (blue), PSU (red) and the PANI-coated PSU (black). The PANI spectrum shows the usual pattern of benzenoid and quinoid stretches. Specifically, the C=C stretches of quinoid and benzenoid are present at 1560 cm^−1^ and 1480 cm^−1^, respectively. The FTIR results show the characteristic bands for the polysulfone backbone. Both PANI-coated PSU and neat PSU membranes have very similar infrared absorption bands which indicate that the fabricated membrane sensors have the same basic structure of PSU support membrane. The broad and weak stretch around 3450 cm^−1^ indicates the presence of the phenolic end group (OH). The other absorption bands are in good agreement with standard polysulfone: 3010 cm^−1^ (aromatic CH), 2880 cm^−1^ (aliphatic CH), 1290 cm^−1^ (S=O), 1240 cm^−1^ (C-O), and 1150 cm^−1^ (C-(SO_2_)-C) peaks Interestingly, the S=O and C-O stretches of the PSU backbone at 1290 cm^−1^ and 1150cm^−1^ are reduced in the PANI-coated PSU spectrum possibly due to hydrogen bonding interactions with polyaniline Emeraldine Salt (ES) form.

### 3.4. SEM Analysis

**Figure 2:**
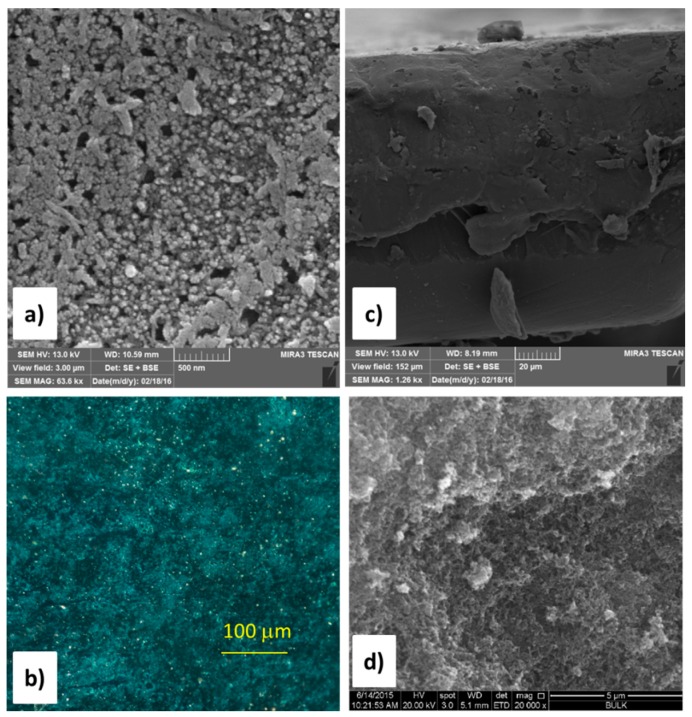
SEM images for the surface of PANI-PSU-20 (**a**); cross-section of the same membrane PANI-PSU-20 (**c**); and polyaniline nanofibers collected from the same reaction (**d**); Optical images in the dark field mode for PANI-PSU-20 (**b**).

The successful coating of polyaniline on the top of PSU membranes is evidenced from the SEM images for the surface of the PANI-coated PSU membranes (Figure 2). The polyaniline top coating is characterized by porous and rough surface morphology as shown in Figure 2a. Further conformation for the porous structure of polyaniline coating can be revealed from the optical micrograph, (Figure 2b). Polyaniline nanofibers possess advantages such as small nanofiber diameter, larger surface area compared with traditional agglomerated polyaniline and the ability to form porous structures when deposited as thin films [48,53]. The porous nanostructure enhances the diffusion of molecules and dopants into the nanofibers which improves the sensitivity, response time and dynamic performance of the fabricated sensor [53,54,55]. Although the morphology of the polyaniline cannot be observed directly from the SEM images of the deposited film, we found that the precipitated polyaniline collected from the same reaction exhibits nanofibrillar morphology, (Figure 2d). The nanofiber morphology could be attributed to the following two factors: (1) the fast mixing of APS initiator and aniline monomer which helps to suppress the secondary growth of agglomerated particles [56]; and (2) the dilute concentrations used for both monomer and initiator [48]. The thin coating of polyaniline is clearly shown in the cross-sections of PANI-coated PSU membranes samples, (Figure 2c).

### 3.5. Performance of PANI-Coated PSU Membranes as Optical pH Chemical Sensor

**Figure 3:**
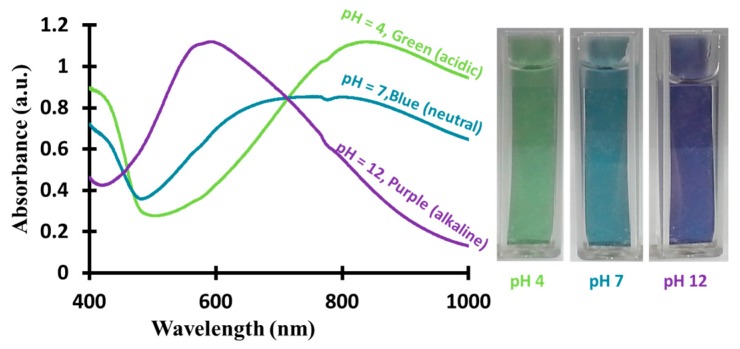
Digital images for optical membrane sensor immersed in solutions with different pH (**right**); and the corresponding visible-NIR absorption spectrum (**left**).

**Figure 4:**
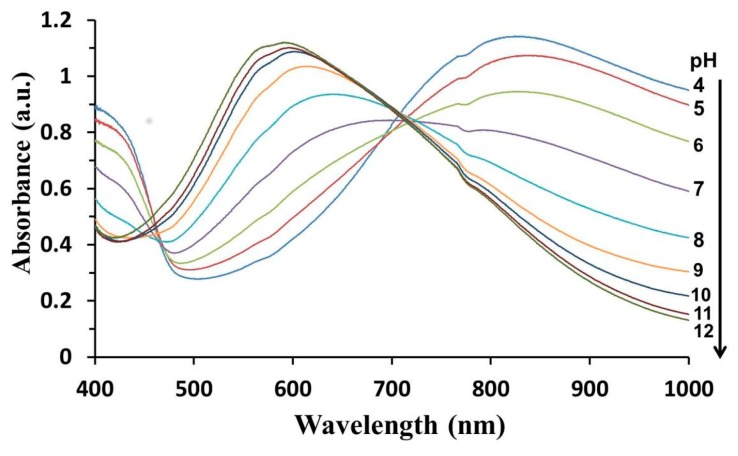
Absorption spectra of the PANI-PSU-20 membranes at different pH.

**Figure 5:**
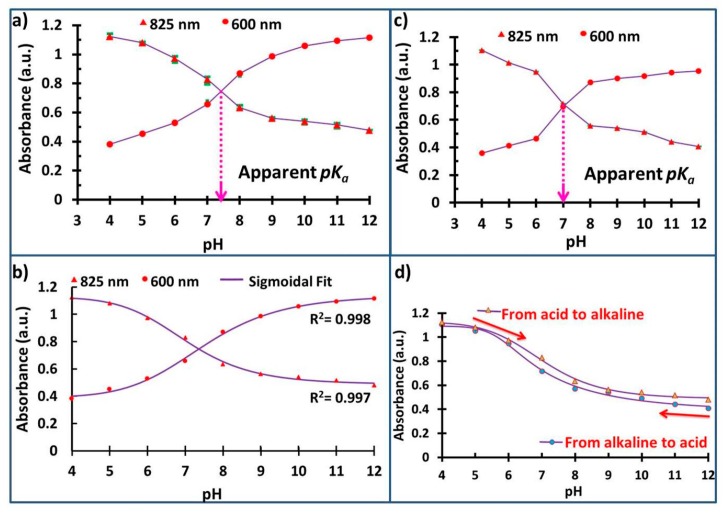
Graphs of the absorbance change vs. pH for PANI-PSU-20 membranes during forward titrations, pH 4 → pH 12, (**a**); and backward titrations, pH 12 → pH 4, (**c**); Dual wavelength calibration curves for the pH dependence of the absorption at 600 and 825 nm (**b**); and the pH titration curves demonstrating hysteresis at 825 nm (**d**).

As shown in Figure 3, membranes with the pH-responsive polyaniline coating exhibit interesting color changes in different environments (acidic, neutral and alkaline) which allow for the determination of pH over a wide dynamic range. The prepared sensor membranes were tested as an optical pH sensor across the visible and near infra-red (NIR) regions (400–1000 nm). PANI-coated PSU membranes exhibit well-defined pH sensitivity in the pH range of 4–12 (Figure 4). The experiments were conducted from pH 4 to 12, with 1 pH unit increments. After each experiment, the coated membrane was rinsed twice with distilled water, followed by one time rinse with 1 M HCl, and finally was placed in the new pH buffer solution and kept for 1 min before measurement in order to obtain comparable results for all the acquired spectra. It is important to note that rinsing the membranes with 1 M HCl provided reproducible results between different runs when moving from low to high pH values or vice versa. This experimental procedure was developed based on the previous literature findings which suggested the storage and preconditioning of PANI-coated sensors in acidic solutions (e.g., pH = 2) [36,38,47]. We have selected 1 M HCl as re-doping solution due to the fact that: (1) the polymer was synthesized using 1 M HCl as dopant; (2) the re-doping process will be faster as the 1 M HCl solution has pH = 0; and (3) 1 M HCl solution does not have extra interfering species that are available in buffer solutions. The re-doping process helps to bring back the polyaniline to its original fully doped acidic state, and accordingly, the starting point is the same when moving between different experiments. As can be inferred from Figure 5, increasing the pH from 4 to 12 led to shift in the absorption **λ**_max_ of polyaniline from 825 nm (at pH 4) to 600 nm (at pH 12). The protonated form of polyaniline Emeraldine Salt (ES) has green color in acidic solutions, which turns into purple colored deprotonated polyaniline Emeraldine Base (EB) in alkaline solutions via doping/de-doping process, (Scheme 1) [44,54].

### 3.6. pH and pK_a_ Measurements

The calibration graph for pH dependence of the absorptions at 600 and 825 nm is displayed in Figure 5a. The measurements were done in triplicates with a standard deviation of the absorbance value over the calibration range between 0.002 and 0.05. The obtained data were fitted by using the four parametric logistic Equation (1), and the sigmoid shape calibration curve (R^2^ = 0.997) was obtained for the absorbance change vs. pH, Figure 5b. The results are similar to the previously reported results for polyaniline coatings [38,57,58]. The broad calibration curve allows for application of the polyaniline sensor membrane over a wide range of pH which is not possible for common pH indicator dyes. The apparent *pK*_a_ is defined as the pH where response is half way between the minimum and maximum values, representing a distribution of the *pK*_a_s of polyanilines with varied chain length and different structural moieties [52]. In the current study, the apparent *pK*_a_ value of the PANI coating is ≈7.35, representing the pH value where the two sigmoidals intersect (concentration of ES is approximately equal with the concentration of EB; Figure 5a). The same value was obtained using PSU membranes coated with polyaniline for 10 min reaction time [59]. A very close *pK*_a_ value ≈7.38 was obtained for polyaniline films coated in the inner walls of polystyrene cuvettes [57]. Florea et al. [39] reported the absorbance vs. pH dependence curve with similar sigmoidals pattern for the PANI-coated optical pH sensor integrated into microfluidic device, with apparent *pK*_a_ value of ≈5. In the former report, the ratio of initiator: monomer was (0.25:1), which is five folds lower than the ratio used in the current study (1.25:1). Polyaniline-polyvinyl alcohol (PANI-PVA) composite membranes prepared by dispersion polymerization showed an apparent *pK*_a_ value of ≈4 [36]. The variation of the apparent *pK*_a_ values could be attributed to the difference in the synthetic procedures and stoichiometric ratio of monomer: initiator which will result in varied molecular weight, molecular structure and surface morphology of the incorporated polyanilines [60].

### 3.7. Hysteresis

The hysteresis behavior of polyaniline-based optical pH sensors have been reported in different studies [36,47,52]. Hence, it is important to address the effect of the hysteresis on the performance of the fabricated sensor membrane during the practical pH measurements. Figure 5c displays the pH dependence of the absorptions at 600 and 825 nm during the back titrations from pH 12 to pH 4. Although the constructed curve in Figure 5c follows a similar sigmoidal pattern, the hysteresis behavior of the sensor membrane can be understood from the small variation in the obtained *pK_a_* value during the back titrations (≈7.0) compared to that *pK_a_* value obtained during the forward titrations (≈7.35). The slight hysteresis in the pH titrations curves shown in Figure 5d suggests that the measured responses during the forward titrations (de-doping process) are higher than those responses recorded during the back titrations (doping process). A similar hysteresis behavior was reported for polyaniline films coated on glass substrates [47]. The observed hysteresis could be attributed to the difference in the swelling degree of polyaniline at different pH values [36] which leads to a change in the porosity and conformation (coiled/stretched) of polyaniline during various doping and de-doping processes [47,52]. Regardless of the observed hysteresis during the pH titrations, the prepared sensor membranes still can be used with reproducible results by conditioning the polyaniline film prior to each new measurement. Indeed, the preconditioning step in 1 M HCl provides quick, practical and reliable method to overcome the limitations of the hysteresis. Hence, the calibration curve shown in Figure 5b can be used for deducing the pH values based on the measured absorbance. For applications that require continuous pH monitoring, a proper calibration curve which considers the movement direction, from low to high pH, or vice versa has to be established.

### 3.8. Response Time

**Figure 6:**
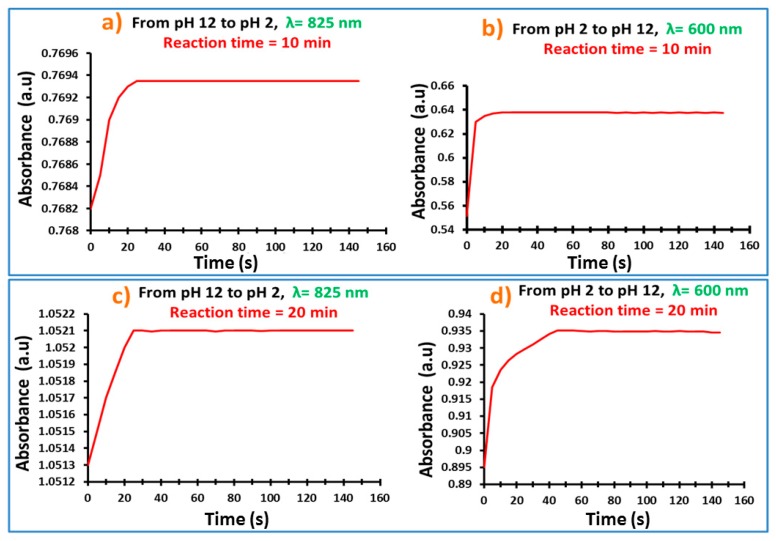
Response time for PANI-PSU-10 membranes (**a**,**b**) and PANI-PSU-20 membranes (**c**,**d**). Each membrane was placed in a buffer solution of pH 12 and then in buffer solution of pH 2 for the measurement of response time at 825 nm; after that, the same membrane was placed back in a buffer solution of pH 12 for the measurement of response time at 600 nm.

Response time represents an important feature for the performance of a specific sensor. The sensor response time is defined as the time required for the sensor output to reach 90% of the change from its previous value to the final settled value [25]. The response time of PANI-coated membranes was measured for the transitions from pH 12 to pH 2 at 825 nm and from pH 2 to pH 12 at 600 nm (Figure 6). Although the response time for both PANI-PSU-10 and PANI-PSU-20 is almost identical, a careful look to the graphs shows that PANI-PSU-10 membranes reached a maximum and steady-state absorbance in shorter times. This behavior could be attributed to the faster equilibrium time for PANI-PSU-10 membranes bearing lower thickness of polyaniline coating compared to PANI-PSU-20 membranes. Nonetheless, all membranes reached >98% of the maximum recorded absorbance within less than 5 s. The fast response times could be attributed to the nanofibrillar morphology of polyaniline with higher surface area presented by the small nanofiber diameter and porous nature of the nanofiber film [53,61].

### 3.9. Storage, Stability and Reproducibility of PANI-Coated PSU Sesnor Membrane

**Figure 7:**
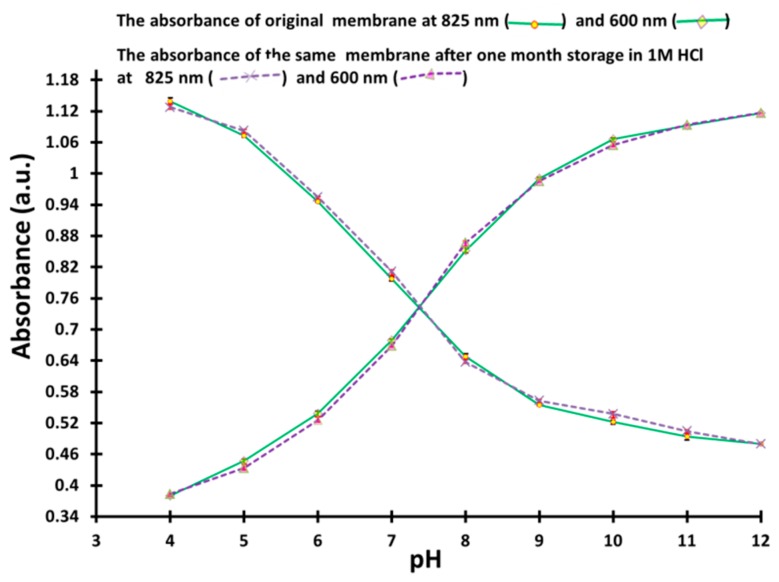
Performance of PANI-PSU-20 membranes after 1 month storage in 1 M HCl. Error bars indicate the standard deviation of absorbance for three replicate samples.

All membranes were stored in a 1 M HCl solution for more than 6 months without any observed mechanical disintegration/deterioration of the stored membranes. The measured response of the tested PANI-PSU-20 membranes after a one-month storage period in the 1 M HCl solution was found to be consistent with <0.02 change in the recorded absorbance values (Figure 7). Hence, PANI-PSU-20 membranes showed insignificant change in their response behavior providing reproducible results over the pH range of 4–12 even after storage for long periods in 1 M HCl solutions.

### 3.10. Real Sample Analysis

The fabricated optical pH sensor membrane was tested for analysis of few samples. Sodium bicarbonate, sodium carbonate and Dettol detergent samples were selected and the results for these samples were compared with results obtained using a commercial electrochemical pH meter (Table 2). The measurements were carried out in triplicates using the same membrane for the three samples. The membrane was preconditioned in 1 M HCl between different measurements. The calibration curve displayed in Figure 5b was used and the pH values were determined accordingly by applying Equation (2). The detection results obtained from the optical pH sensor membrane are in good agreement with those obtained using the commercial electrochemical pH meter. It is worth mentioning that the tested Dettol detergent sample contains a mixture of organic molecules/solvents (e.g., isopropyl alcohol, chloroxylenol, pine oil) and yet the performance of the optical pH sensor was not compromised likely due to the good chemical stability of both polyaniline coating and PSU support matrix.

## 4. Conclusions

The in situ COP of polyaniline provided a simple, quick, cheap and precise way to construct an optical pH sensor membrane with controlled thickness and morphology of polyaniline coating. Polyaniline coatings with thicknesses of ≈100–200 nm were obtained and tested as optical pH sensors in the pH range of 4–12. A reaction time of 20 min provided response signal of ≈1.1 absorption units. The optical pH sensor was characterized by fast response time (<4 s) which could be attributed to the enhanced diffusion of dopants through the thin polyaniline coating with rough porous and nanofibrillar morphology. A dual calibration curve was constructed using the maximum absorbance at 600 nm and 825 nm for different pH solutions. The obtained sigmoidal calibration curve (R^2^ = 0.997) was used for deducing the pH values for different samples. The obtained pH values using the optical pH sensor were in good agreement with those results obtained using an electrochemical pH meter.

The fabricated pH sensor membranes are flexible and exhibit well-defined and fast pH responses in the pH range of 4–12. The optical pH sensor membranes can be stored in 1 M HCl solutions for long periods without performance deterioration and with consistent reproducibility behavior. The observed small hysteresis can be overcome by a preconditioning step prior to each new measurement. Future work will focus on the correlations between performance of the optical pH sensor with both morphology and thickness of polyaniline coating in order to obtain more consistent readings without remarked hysteresis for both forward and backward pH titrations.

## Figures and Tables

**Table 1:** Reaction conditions during the COP. Molar concentration of aniline was fixed to (0.05 M). The molar ratio of (aniline: APS) was fixed to (1:1.25). All reactions were carried out at room temperature using 1 M HCl as solvent.

Membrane Code	Reaction Time (min)	Absorbance at pH 4 (λ = 825 nm)	Estimated Coating Thickness ^1^
			(nm)
PANI-PSU-10	10	0.63	102 ± 7
PANI-PSU-20	20	1.08	160 ± 9

^1^ The thickness of the polyaniline coating was estimated using Equation (3). The absorbance was measured in 1 M HCl solutions.

**Table 2:** pH detection for different samples using the optical pH sensor compared with a commercial pH meter.

Sample	Color	pH Sensor	pH Meter	% Error ^1^
0.10 M NaHCO_3_	Colorless	8.63 ± 0.01	8.34 ± 0.01	3.48
0.10 M Na_2_CO_3_	Colorless	11.12 ± 0.06	11.37 ± 0.01	2.20
Commercial Dettol Detergent	Transparent Brown	9.58 ± 0.03	9.68 ± 0.02	1.03

^1^ %Error = ((pH sensor reading − pH meter reading)/ (pH meter reading)) × 100.
